# Traumatic Events, Personality and Psychopathology in Takotsubo Syndrome: A Systematic Review

**DOI:** 10.3389/fpsyg.2019.02742

**Published:** 2019-12-10

**Authors:** Federica Galli, Francesca Bursi, Stefano Carugo

**Affiliations:** ^1^Cardiology Unit and UCIC, UOC Cardiology, ASST Santi Paolo e Carlo, S. Paolo Hospital, Milan, Italy; ^2^Department of Health Sciences, Università di Milano, Milan, Italy

**Keywords:** Takotsubo syndrome, psychological factors, life-event, trauma, anxiety, depression, personality

## Abstract

**Objective:**

Takotsubo syndrome (TTS) is a transient heart disease that has been historically related to the occurrence of psychological (emotional) factors (“broken heart” syndrome). We aimed to conduct a systematic review analyzing the role of psychological factors in TTS.

**Methods:**

All studies on TTS and psychological factors from January 1991 through April 2019 were scrutinized according to the Cochrane Collaboration and the PRISMA statements. Selected studies were additionally evaluated for the Risk of Bias according to the Newcastle-Ottawa Scale (NOS).

**Results:**

Fifteen case-control studies (by Mayo Clinic criteria) were finally selected. Most studies analyzed stressful life-events or trauma, although with conflicting findings, while a likely role of long-lasting psychological distress seemed to be a homogenous result. Among life-time psychopathology, only anxiety appeared to have a significant role. Some studies outlined a likely role of personality, but findings are conflicting.

**Conclusion:**

Our findings do not lead to any definitive assumption on the specific role of psychological factors in TTS, also for scant strong methodology of the most part of the studies. More studies with stronger research methodology are needed to better characterize psychological elements in TTS.

## Introduction

Takotsubo syndrome (TTS) is a form of transient heart failure syndrome often mimicking acute myocardial infarction. It is also known as stress cardiomyopathy, broken heart syndrome, or apical ballooning syndrome, and was firstly described in 1990 (Dote et al., 1991), even if some Authors dated it earlier (Wittstein, 2008).

Takotsubo syndrome is characterized by acute, but reversible, left ventricular regional systolic dysfunction accompanied by electrocardiographic changes and cardiac biomarkers elevation in the absence of a significant pathological condition (Wittstein, 2008). The Position Statement from Heart Failure Association of the European Society of Cardiology has published new TTS diagnostic criteria (Parodi et al., 2014). The estimated prevalence of TTS is about 1–3% of all patients presenting with suspected acute coronary syndrome and up to 5–6% in female patients (Ghadri et al., 2018), indeed it is predominantly observed in postmenopausal women and elderly (Templin et al., 2015). The pathophysiological mechanisms responsible for TTS are complex and may vary between patients (Agewall et al., 2017) The prognosis is generally good (Agewall et al., 2017).

Since the first descriptions of TTS, a role for psychological factors has been underscored in medical literature (Cebelin and Hirsch, 1980; Pavin et al., 1997; Prasad et al., 2008), as testified by the previously used terms “stress” cardiomyopathy or “broken-heart” syndrome to name TTS. Clarifying the role of psychological factors in TTS may add new framework issues and possibilities of intervention for these patients. The recent Consensus document on the diagnostic workup and management of TTS (Ghadri et al., 2018) outlines a specific role (diagnostic algorithm) of psychopathologic disorders and emotional stress for the diagnostic workflow of patients in the emergency department. However, evidence on the role of psychological factors in TTS are sparse, and mainly related to case-reports (Wang et al., 2015; Manfredini et al., 2018).

To the best of our knowledge, no systematic reviews have been realized analyzing the likely causative link between psychological factors and TTS, with the existing reviews detecting other factors (e.g., the role of drugs or pathophysiologic mechanisms). The objective of the present study was to systematically review all the studies on psychological factors (as antecedents) (traumatic/stressful events, psychopathology and personality) in patients with TTS diagnosis in order to understand their likely role in this syndrome.

## Methods

We conducted a systematic review of the literature on psychological factors (psychopathologic disorders, stressful life-events/psychological trauma, and personality characteristics) in TTS. All observational studies were included in the review by the ascertainment of a case-control study design, adequacy of the sample size, comparison, and outcome measures.

### Search Strategy

To include the broadest range of relevant literature, electronic searches were conducted on the major databases in the field of health and social sciences: Pubmed, Scopus, Embase, PsycInfo, and Web of Science. The search was performed using Mesh terms OR Keywords (depending on the database) with the same search strategy: “Takotsubo ” OR “Tako-Tsubo syndrome” OR “Stress – induced cardyomiopathy” OR “Takotsubo cardiomyopathy” “transient left ventricular ballooning syndrome” OR “apical ballooning syndrome” OR “ampulla cardiomyopathy” OR “broken heart syndrome” AND “Psychological distress” OR “Anxiety” OR “Depression” OR “Emotional distress/trigger” OR “acute stress” OR “Personality” OR “Psychiatric disorder” OR “Temperament” OR “Life-event”. The selection of the search terms was based on the clinical experience and the literature topics on psychological factors involved in physical disorders (American Psychiatric Association [APA], 2013). The search was limited to English-written publications, and to the period from January 1991 to April 2019. An additional analysis of the reference list in each selected paper was also performed. When the full text was not retrievable, the study was excluded.

### Selection Criteria

#### Inclusion Criteria

• Studies with an analytical study design as defined by Grimes and Schulz (2002) (i.e., an observational study with a comparison or control group Both retrospective and perspective studies have been included to consider the highest number of studies.
• Diagnosis of TTS by the Mayo Clinic criteria or by the new TTS criteria (Parodi et al., 2014; Lyon et al., 2016).
• Studies adopting standardized and validated tests.
• Studies written in English language.

#### Exclusion Criteria

• Studies with intra-group control (e.g., TTS with a pre-existing disorder or not).
• Case reports, reviews, Letters to the Editor, meeting abstracts, book chapters.
• Pharmacological and behavioral intervention trials, surgical protocols, or validation of measurement instruments.
• Number of subjects per group ≤ 5.

### Data Extraction

Study selection was performed by two independent reviewers with research expertise in clinical psychology and cardiology (FG and FB) who assessed the relevance of the study for the objectives of this review. This first round of selection was based on the title, abstract, and keywords of each study. If the reviewers did not reach a consensus or the abstract did not contain sufficient information the full text was reviewed.

**FIGURE 1:**
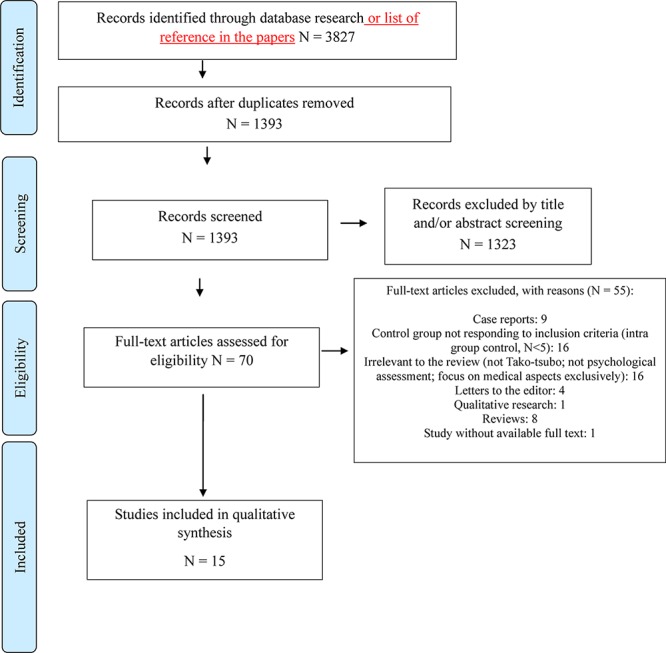
PRISMA flow diagram of literature search and selection of publications.

In the second phase (screening), full-text reports were evaluated to detect whether the studies met the inclusion criteria (Figure 1).

In the phase of eligibility, all full-texts were retrieved and a final check was made to exclude papers not responding to inclusion/exclusion criteria, and reaching the final consensus to decide the final number of studies to be selected.

A standardized data extraction form was prepared; data were independently extracted by two of the authors (FG and FB) and inserted in a study database. A process of discussion/consensus moderated by a third reviewer (SC) (Furlan et al., 2009) resolved discrepancies between reviewers.

### Statistical Methods

A systematic analysis was conducted according to the Cochrane Collaboration guidelines (Higgins and Green, 2011) and the PRISMA Statement (Moher et al., 2009). Because the included studies were highly heterogeneous in terms of participants, variables, instruments, and outcomes, it considered inappropriate to undertake a meta-analysis (Higgins and Green, 2011). However, effect size computations were performed using Cohen’s *d* (Cohen, 1988) and its 95% confidence interval for continuous variables for each outcome measure within each study (Borestein et al., 2011). The index was primarily calculated using descriptive statistics reported in the results section of each study. When binary data was reported, we estimated the Odds Ratio with related estimates of confidence intervals (Borestein et al., 2011). Cohen’s *d* values less than or equal to 0.20, 0.50, and 0.80 were interpreted as small, medium and large effect size, respectively (Cohen, 1988). For variables expressed as median and 25th–75th percentile it was not possible to calculate Cohen’s *d*.

### Risk of Bias

Quality assessment of each of the included studies was evaluated following the Newcastle-Ottawa Scale (NOS) for case-control studies on a 9-star model (Wells et al., 2014). Studies scoring above the median NOS value were considered as high quality (low risk of bias) and those scoring below the median value were considered as low quality (high risk of bias). In brief, two reviewers (FB and FG) independently extracted relevant information and data from all eligible reports that met the above inclusion criteria.

## Results

We found 15 studies meeting inclusion criteria (Figure 1), for a total of 2581 subjects (1152 TTS patients; 1069 other heart diseases; 360 healthy controls). The description of the samples, psychological variables, study design, psychometric scales (tests, interview or retrospective medical records analysis), key findings, Cohen’s *d* or Odd ratios and 95% confidence intervals of selected studies are reported in Table 1.

**TABLE 1:** Overview of the selected studies.

**Study**	**Population**			**Study characteristics**			**Significant findings (p)**	***Cohen d* (95% CI)**	**Notes**	**Nos Score**
	**TTS (*N*, mean age, *SD*), *N* females (f)**	**Controls (*N*, mean age, *SD*), *N* females (f)**	**Control (diagnosis)**	**Assessment (time after diagnosis)**	**Outcome measures**	**Design**				
Del Pace et al., 2011	50 (73 ± 9); 47f	50 (73 ± 9), 47f	STEMI	– Before hospital discharge	– STAI	Prospective case-control	– Stressful trigger event: TTS > STEMI (*p* < 0.001)	STAI *d* = 0.08 (−0.32–0.47) – Stressful trigger event OR 46.0 (13.38–158.09)	– High anxiety trait common to TTS and STEMI – Unclear how the antecedent stressful trigger event has been evaluated	5/9
Delmas et al., 2013	45 (72.7 ± 11.5); 41f	50 (72.9 ± 13.1); 47f	– ACS	– Within 48 h after admission	– Mini interrnational neuropsy- chiatric interview	Prospective case-control.	– ADD: TTS > ASC (*p* < 0.001) – Acute stressful event: 78% (TTS) vs. 18% (ACS) – Chronic psychological stress: TTS > ACS) (*p* = 0.005) – Current and/or past MDD: TTS > ACS (*p* < 0.001)	– TTS vs. ACS ADD OR 9.96 (3.87–25.63) – Acute stressful event OR 15.94 (5.82–43.65) – Chronic psychological stress OR 3.64 (1.44–9.24) – Current and/or past MDD OR 7.83 (3.13–19.52)	– ADD more common in TTS than ACS – Unclear as the acute stressful event and chronic psychological stress has been evaluated	4/9
Compare et al., 2013	37^∗^ (66 ± 12.8), 33f 38^∗∗^ (66 ± 11.1),34f	37 (66 ± 10.1), 33f	– AMI	– Within 48 h of symptoms (emotional symptoms assessment) – 3 months after symptoms (personality assessment)	– IRLE – PSI – DS14 questionnaire (Type D personality)	Cross-sectional, prospective case-contro.	– Type-D Social Inhibition sub-scale: TTS > AMI (p < 0.001)	– *d* = 5.3 (4.3–6.3) tTTS vs. nt TCC – *d* = 5.05 (4.1–6.1) tTTS vs. AMI	– No differences in emotional triggering events (TTS vs. AMI)	5/9
Compare et al., 2014	37 (55 ± 8), 33f	37 (57 ± 7), 33f	– AMI	– During first visit following admittance (by cardiologist) – 1-year follow-up (by clinical psychologist)	– IRLE – PSI – MacNew – PGWBI	Prospective case-control (1-year follow-up)	– Psychological distress TTS > AMI (*p* = 0.001) (1-year follow-up)	– Baseline TTS vs. AMI: PGWBI *d* = 0.02 (−0.44–0.49) – MacNew global *d* = −0.20 (−0.66–0.26) – 1 year TTS vs. AMI – PGWBI *d* = 2.72 (2.07–3.37); MacNew-global *d* = 1.91 (1.35–2.47)	– No patients with TTS had a “history of psychiatric illness” – The psychological distress in TTS tends to become more negative over time than in AMI	7/9
Kastaun et al., 2014	19 (61.1 ± 9.5)	20 (62.2 ± 10.0) 20 (58.4 ± 7.8)	– NSTEMI myocardial infarction – HV	– 18.4 (+8.5) months from TTS and NSTEMI acute event – Acute stressful event from medical record	– SCL-90 – FPI-R – TICS – MDMQ	Prospective case-control	– 2 (11%) TTS vs. 12 (60%) NSTEMI showed no acute trigger event (*p* = 0.014) – Life event inventory (physical/sexual abuse, traumatic experience): 8 TTS (42%) vs. 2 NSTEMI (10%) vs. 2 HV (10%) (*p* = 0.031) – TTS differed from controls for: FPI-R greater emotionality (*p* = 0.048); TICS chronic worrying (*p* = 0.030). – TTS: lower overall cortisol release (p < 0.05) – TTS felt significantly more nervous than controls at arrival (*p* < 0.01), post-stress (*p* < 0.05) and the end (<0.05) of examination	– No acute trigger OR 0.08 (0.01-0.44) TTS vs. NSTEMI – Life event inventory (physical/sexual abuse, traumatic experience): OR 6.54 (1.17-36.61) TTS vs. NSTEMI and HV.	– The psychological assessment and cortisol measure have been released 18 months after the cardiac event. – TTS showed lower cortisol release than controls. – No significant differences in SCL-90 scoring. – TTS showed more nervousness during stress situations, but they had lower cortisol level – Limited sample size	6/9
Lacey et al., 2014	31 sporadic TTS (sp-TTS) (66.7) 27 earthquake TTS (eq-TTS) (69.7)	26 (80.9)	HV	– In 12 months following the earthquake – From hospital records (2000–2012)	– EPQ-brief	Retrospective case-control	– Past experience of trauma connoted TTS (59% eq-TTS vs. 42% sp-TTS vs. 23% HV, *p* < 0.05) – EPQ neuroticism > in eq-TTS and sp-TTS vs. HV (*p* < 0.01)	– Past experience of trauma OR 4.84 (1.47–15.97) eq-TTS vs. HV OR 2.41 (0.76–7.67) sp-TTS vs. HV – EPQ extraversion *d* = −0.25 (−0.80–0.30) eq-TTS vs. HV; *d* = −0.28 (−0.81–0.25) spTTS vs. HV – EPQ neuroticism *d* = 1.34 (0.73–1.95), eq-TTS vs. HV; *d* = 1.04 (0.48–1.60) sp-TTS vs. HV	– The timing of assessment is very different in the three groups (also 10 years and more) – Antecedent psychiatric factors do not distinguish the three groups – Mean age of healthy volunteers is much higher than TTS – The semi-structured interview is not validated and the assessment criteria have not been described at all	7/9
Templin et al., 2015	455 (67.7 ± 12.5), 411f	455 (68.7 ± 12.3), 411f	– ACS	– Review of medical records (presumably during index hospitalization, but unspecified)	– DSM-IV	Multicentre retrospective case-control	– Acute, former or chronic neurologic *or* psychiatric diseases TTS > ACS (*p* < 0.001) – Total psychiatric disorders: TTS > 14.3% (*p* < 0.001) – Acute psychiatric disorders (12.6% TTS vs. 1.3% ACS) – Former or chronic psychiatric disorders TTS > ACS (*p* < 0.001); – Former or chronic affective disorders TTS > ACS (*p* < 0.001); – Acute affective disorders TTS > ACS (*p* = 0.002)	– Acute, former or chronic neurologic *or* psychiatric diseases OR 3.65 (2.75–4.83) – Total Psychiatric disorders OR 4.39 (3.17–6.07) – Acute psychiatric disorders OR = 10.6 (4.53–24.9); – Former or Chronic psychiatric disorders OR 3.66 (2.62–5.10) – Former or chronic affective disorders OR 3.05 (2.03–4.63); – Acute affective disorders OR 12.2 (1.58–94.15) – Former or chronic anxiety disorder OR 12.3 (4.39–34.51)	– Higher prevalence of psychiatric (*or* neurologic) disorders in TTS history – The psychiatric diagnosis has not been made by psychological test (it is presumably clinical according to DSM-IV criteria). – The distinction between former and chronic is not clear (not by DSM-IV criteria)	3/9
Christensen et al., 2016	45 (70)	95 (72) 90 (67)	– STEMI – HV randomly selected by Danish civil registry	– 24 months (min 8-max 36) for TTS – 26 months (min 2-max 32) for STEMI	– WHO-5 Well-Being Index – ENS – MDI – ASS (anxiety sub-scale)	Prospective case-control	– Anxiety score higher in TTS vs. STEMI (*p* = 0.007) – Compared to control group, TTS: less well- being (*p* = 0.02); higher neuroticism (*p* = 0.0002); more depression (*p* = 0.007); more anxiety (*p* = 0.0001) – Well-being: TTS < STEMI: (*p* = 0.0009); neuroticism TTS > STEMI(*p* = 0.01); depression: TTS > STEMI (*p* = 0.006); anxiety (ns)	Median values	– Only the level of anxiety differentiated TTS and STEMI – Tests mailed	5/9
Goh et al., 2016	73 (67.7 ± 9.8),71f	111 (68.2 ± 10.2), 109f	– ACS (with cardiac catherization)	– 33.8 months (TTS) – 36.3 months (ACS)	– HADS	Retrospective case-control.	– Anxiety HADS score: TTS vs. ACS (*p* = 0.06) – Emotional stress as trigger in 43%TTS vs. 23%.	– HADS *d* = 0.32 (0.02–0.61) – Emotional stress as a trigger OR 4.08 (2.04–8.17);	– The time range of HADS administration is very large – A role for anxiety may not be supported by data – Triggers in ACS group had not been detected.	5/9
Salmoirago-Blotcher et al., 2016	45 (62.4 ± 10.8)	32 (64.6 ± 15.3) 30 (55.9 ± 12.4)	– MI (24 STEMI and 8 NSTEMI) – HV	– 1 month after hospital discharge (telephone interview)	– HADS – PSS – IES – Cook-Medley hostility inventory – Life orientation test – DS14 questionnaire (Type D personality) – PERI Life (adapted)	Prospective case-control and cross-sectional design	– Psychiatric disorders: anxiety > TTS (24.4%) > MI(12.5%) > HV (0%) (*p* = 0.01) – Psychological distress (HADS): TTS vs. MI (ns); TTS > HV (*p* < 0.05) – PTSD: TTS > MI > HV (*p* < 0.001) – PSS: TTS > HV (*p* < 0.05) – Personality (negative affectivity): TTS > HV (*p* < 0.05) – Personality (social inhibition): TTS > HV (*p* < 0.05) – Stressful life events: TTS > HV (*p* < 0.001); TTS vs. MI (ns)	– Anxiety OR 2.27 (0.64-7.90) TTS vs. HV – HADS *d* = 0.2 (-0.26-0.67) TTS vs. MI, *d* = 0.75 (0.27-1.22) TTS vs. HV – PS score *d* = 0.34 (−0.12–0.80) TTS vs. MI, *d* = 0.66 (0.20–1.17) – IES score *d* = 0.54 (*d* = 0.07–1.00) TCC vs. MI *d* = 1.00 (0.51–1.50) TTS vs. HV – Type D personality negative affectivity *d* = 0.11 (−0.35–0.57) TTS vs. MI, *d* = 0.64 (0.16–1.12) TCC vs. HV – Type D personality social inhibition *d* = 0.10 (−0.35–0.57) TTS vs. MI *d* = 0.66 (0.18–1.13) – Stressful life event OR = 1.33 (0.45–3.94) vs. MI; OR = 4.57 (1.64–12.72) TCC vs. HV	– TTS: higher level of pre-discharge anxiety and post discharge psychological distress. – Personality did not differ comparing TTS and MI. -Data on psychiatric disorders have been drawn retrospectively from the medical records.	9/9
Smeijers et al., 2016	18, 14f (68.3 ± 11.7)	19, 13f (68.8 ± 10.1) 19 HV, 13f (60 ± 7.6)	– chronic HF – HV	– 23 months (+18)	– PHQ-9 – PSS – GAD-7 – Whiteley-7 scale (anxiety) – NEO-FFI	Prospective case-control.	– depression: TTS > HV (*p* < 0.05); – illness-related anxiety: TTS > HV (*p* < 0.01). – Level of openness: TTS < HV (*p* = 0.021).	– TTS vs. HV PHQ *d* = 0.67 (−0.02–1.36); – PSS *d* = 0.61 (−0.08–1.3); GAD *d* = 0.52 (−0.17–1.20); – Whiteley-7 scale *d* = 0.92 (0.21–1.65); – NEO FFI openness *d* = −0.8 (−1.50–0.99)	– TTS patients did not differ from HF for any of the psychological measures	5/9
Rosman et al., 2017	45 (62.4 ± 10.8)	32 (64.6 ± 15.3) 30 (55.9 ± 12.4)	– MI (24 STEMI and 8 NSTEMI) – HV	– 1 month after hospital discharge (telephone interview)	– PERI Life (adapted)	Prospective case-control.	– TOTAL number of events (along 6 months): TTS > MI > HV (*p* < 0.001). – anxiety: TTS > MI and HV (*p* = 0.007)	– Total N events *d* = 0.62 (0.15–1.10) TCC vs. MI, *d* = 1.21 (0.70–1.73) TCC vs. HV – Stressful event within 1 year prior to hospitalization OR = 0.89 (0.33–2.32) TTS vs. MI – Anxiety OR 3.12 (0.79–12.30)	– Not the event of the month before hospitalization, but the sum of multiple events during the last 6 months were linked to TTS	5/9
Saffari et al., 2017	94 (63.56 ± 7.48)	94 (62.8 ± 8.33) 94 (63.1 ± 6.45)	– AMI – HV	– Before discharge – 6 and 18-month follow-up	– FSFI – FSDS-R – Healt Related – HRQoL-SF-12 – HADS	Prospective case-control.	– Sexual distress, anxiety and depression: TTS > AMI > HV (*p* < 0.05) – Worse sexual functioning: TTS > AMI and HV (at 6 and 18 month follow-up) (*p* < 0.001)	– Baseline TTS vs. MI – FSDS-R *d* = 0.50 (0.21–0.79) – FSFI *d* = −0.037 (−0.32–0.24) – Depression HADS *d* = 0.04 (−0.25–0.32) – Anxiety HADS *d* = 0.34 (0.05–0.63) – HRQoL PCS *d* = −0.09 (−0.34–0.19) MCS *d* = −0.52 (−0.81 to −0.22) TTS vs. HV: – FSD-R *d* = 2.4 (2.05–2.81) – FSFI *d* = −1.75 (−2.09 to −1.41) -Depression HADS *d* = 0.64 (−0.243–0.93) – Anxiety *d* = 1.96 (1.61–2.31) – HRQoL: PCS *d* = −3.43 (−3.882.98); MCS *d* = −2.41 (−2.79–2.04)	– Worsening of sexual function and HRQoL across time	7/9
Compare et al., 2018	37^∗^ (66 ± 12.8), 33f 37^∗∗^ (66 ± 11.1),33f	37 (66 ± 10.1), 33f	– AMI^∗^	– 3 months after symptoms	– TMMS – MCQ-30 – EPS – HAM-D	Cross-sectional	– HAM-D: TTS^∗^ vs. TTS^∗∗^ (*p* = 0.004); TTS^∗^ vs. AMI^∗^ (*p* = 0.021); – MCQ-30: TTS^∗^ > AMI and TTS^∗∗^ (*p* < 0.05); – TMMS: TTS^∗^ > AMI^∗^ (*p* < 0.05); – EPS: TTS^∗^ > AMI and TTS^∗∗^ (*p* < 0.05)	– NA	– Emotional competence more compromised in TTS^∗^ than TTS^∗∗^ and AMI^∗^	5/9
Sancassiani et al., 2018	19 (63.26 ± 9.21), 17f	76 (63.26 ± 9.03), 68f	– HV	– 1 month after TTS	– ANTAS – SCID-I/NP	Prospective case-control.	– Any mood disorder: TTS > HV (*p* = 0.002)	– NA	– No evidence of association with anxiety disorders	5/9

*ADD, anxious-depressive disorder; AMI, acute myocardial infarction; ACS, acute coronary syndrome; ANTAS, advanced neuropsychiatric tools and assessment schedule; ASS, Hopkin’s symptoms checklist; ENS, Eysenck’s neuroticism scale; EPQ, Eysenck personality questionnaire; EPS, emotional processing scale; FPI-R, Freiburger personality inventory; FSDS-R, female sexual distress scale-revised; FSFI, female sexual function index; GAD, general anxiety disorder; HADS, hospital anxiety and depression scale; HF, heart failure; HAM-D, hamilton rating scale for depression; HRQoL-SF-12, health related quality of life [physical component summary (PCS) and mental component summary (MCS)]; HV, healthy volunteers; IES, impact of events scale-revised; IRLE, Italian version of the interview for recent life events; MacNew, MacNew heart disease health related quality of life; MCQ-30, meta-cognitions questionnaire; MDD, major depressive disorder; MDI, major depression inventory; MDMQ, multidimensional mood state questionnaire; MI, myocardial infarction; NEO-FFI, NEO five-factor inventory; NSTEMI, non-ST-segment elevation myocardial infarction; PGWBI, psychological general well-being index; PHQ-9, patient health questionnaire; PSI, Paykel stress index; PSS, perceived stress scale; PTSD, post-traumatic stress Disorder; SCID-I/NP, structured clinical interview for DSM-IV axis I disorders/No Patient version; SCL-90, symptom checklist-90; TICS, trier inventory for the assessment of chronic stress; TMMS, trait meta-mood scale; TTS, Takotsubo syndrome; WHO-5, WHO-5 well-being index; QoL, quality of life. ^∗^With emotional stress. ^∗∗^Without emotional stress. NOS score, New Ottawa Scale for case-control study. NB, distinction for gender was inserted when there was a mixed gender sample, otherwise is to be intended that it was a female sample.*

### Life-Events and Psychological Trauma

Most studies investigated the likely role of concurrent stressful events in triggering TTS. The three studies (Compare et al., 2013; Salmoirago-Blotcher et al., 2016; Rosman et al., 2017) investigating the topic by means of psychometric scales did not find any differences between TTS and patients with other cardiac events. Conversely, the studies outlining a significant role of stressful events, collected data by patient interview at admission (Del Pace et al., 2011; Delmas et al., 2013; Compare et al., 2014) or retrospective review of medical records (Kastaun et al., 2014; Templin et al., 2015). One study found a greater impact of Post-Traumatic Stress Disorder (PTSD) after discharge (Salmoirago-Blotcher et al., 2016) in TTS compared to patients with myocardial infarction or healthy controls.

Of interest, the time elapsing between the cardiac event and the psychological assessment was extremely variable, ranging from hours (Delmas et al., 2013) to years (Goh et al., 2016).

History of long-lasting psychological distress not temporally related to the cardiac events was evidenced in four studies (Delmas et al., 2013; Kastaun et al., 2014; Lacey et al., 2014; Rosman et al., 2017).

Some studies differentiated emotional (27–38%) from physical (36–50%) triggers (Compare et al., 2014; Templin et al., 2015; Goh et al., 2016; Rosman et al., 2017), with 12–28% of patients with indeterminable reasons.

### Psychopathology (Lifetime, by Medical Records)

Eight studies described psychopathology only recording data by clinical records (without psychometric tests) in a lifetime perspective (Del Pace et al., 2011; Delmas et al., 2013; Kastaun et al., 2014; Lacey et al., 2014; Templin et al., 2015; Goh et al., 2016; Salmoirago-Blotcher et al., 2016; Rosman et al., 2017). Six studies (Del Pace et al., 2011; Delmas et al., 2013; Kastaun et al., 2014; Lacey et al., 2014; Salmoirago-Blotcher et al., 2016; Rosman et al., 2017) aimed to detect whether a history of psychopathologic factors might be related to TTS. In four studies (Lacey et al., 2014; Templin et al., 2015; Salmoirago-Blotcher et al., 2016; Rosman et al., 2017) the psychopathologic diagnoses were made retrospectively by subsuming clinical data derived from medical records. Among these only one study found a significant association between mood *or* anxiety disorders and TTS (Templin et al., 2015). Two studies (Salmoirago-Blotcher et al., 2016; Rosman et al., 2017) found an association only with anxiety, while one study (Goh et al., 2016) did not find any significant associations.

### Psychopathology (Current, by Standardized Measures)

Eight studies analyzed the presence of past psychopathologic diagnoses by means of psychometric tests after the occurrence of TTS (Del Pace et al., 2011; Delmas et al., 2013; Compare et al., 2014, 2018; Kastaun et al., 2014; Christensen et al., 2016; Smeijers et al., 2016; Sancassiani et al., 2018) with miscellaneous findings. Anxiety *and* depression seemed to be prevalent in the history of TTS in one study (Delmas et al., 2013), while two studies evidenced a role only for anxiety (Del Pace et al., 2011; Christensen et al., 2016). Two studies (Smeijers et al., 2016; Sancassiani et al., 2018) evidenced a role for depression (and not for general anxiety) if compared with healthy controls, but not if compared with patients with chronic heart failure (Smeijers et al., 2016). One study (Compare et al., 2018) compared the prevalence of depression in TTS with emotion triggers vs. acute myocardial infarction with emotion triggers vs. TTS without emotion trigger and found a significant prevalence only in the two groups with emotion triggers.

Other studies (Compare et al., 2014; Kastaun et al., 2014) did not find any role for psychopathology in the history of TTS patients.

### Personality

Five studies (Compare et al., 2013; Kastaun et al., 2014; Lacey et al., 2014; Salmoirago-Blotcher et al., 2016; Smeijers et al., 2016) evaluated the role of personality in TTS. Three studies (Lacey et al., 2014; Salmoirago-Blotcher et al., 2016; Smeijers et al., 2016) found that TTS had pathological characteristics compared to healthy controls, but not if compared to patients with other cardiac events. The remaining studies (Compare et al., 2013; Kastaun et al., 2014) found a greater emotionality and a prevalence of Distressed personality (Type-D, mainly for Social Inhibition) in TTS compared with controls with myocardial infarction. Interestingly, one study (Compare et al., 2018) found a dysfunctional profile in emotional competence in patients with TTS.

### Risk of Bias

Half of the studies reflected the median value (μ = 5), four were above it and three below (Table 2). Four studies were quoted as high quality (low risk of bias) by NOS (see Table 2).

**TABLE 2:** Ottawa-Newcastle risk of bias for case-control studies.

**Authors**	**Selection**				**Comparability^1^**	**Outcome (psychological tests)**			**Total NOS score**
	**Adequate case definition**	**Represen- tativeness**	**Selection of controls**	**Definition of controls**		**Ascertainment**	**Same Ascertainment for case/control**	**Non-response rate**	
Del Pace et al., 2011	^∗^	^∗^	−	^∗^	^∗^	−	^∗^	−	5/9
Delmas et al., 2013	^∗^	^∗^	−	−	^∗^	−	^∗^	−	4/9
Compare et al., 2013, 2018	^∗^	^∗^	−	^∗^	^∗^	^∗^	−	−	5/9
Compare et al., 2014	^∗^	^∗^	−	^∗^	^∗∗^	^∗^	^∗^	−	7/9
Kastaun et al., 2014	^∗^	−	^∗^	−	^∗∗^	−	^∗^	^∗^	6/9
Lacey et al., 2014	^∗^	−	−	^∗^	^∗^	−	−	^∗^	4/9
Templin et al., 2015	^∗^	^∗^	−	−	^∗^	−	−	−	3/9
Christensen et al., 2016	−	^∗^	^∗^	−	^∗^	−	^∗^	^∗^	5/9
Goh et al., 2016	^∗^	^∗^	−	−	^∗∗^	−	−	^∗^	5/9
Salmoirago-Blotcher et al., 2016	^∗^	^∗^	^∗^	^∗^	^∗∗^	^∗^	^∗^	^∗^	9/9
Smeijers et al., 2016	^∗^	−	^∗^	^∗^	^∗^	−	^∗^	−	5/9
Rosman et al., 2017	^∗^	^∗^	^∗^	−	^∗^	^∗^	−	−	5/9
Saffari et al., 2017	^∗^	^∗^	^∗^	^∗^	^∗^	−	^∗^	^∗^	7/9
Sancassiani et al., 2018	^∗^	^∗^	−	^∗^	^∗^	−	^∗^	−	5/9

*^∗^The criterion is reflected in the study. ^1^Two stars were assigned when the control was matched not only for age and gender, but for the index event date as well.*

## Discussion

Although the role of psychological factors has been extensively studied in TTS, only fifteen studies fulfilled the criteria to perform a systematic review. Consequently, we could not perform a meta-analysis, as originally planned, because of the small number of selected studies and the heterogeneous methodology used for the psychological assessment (no studies shared the same psychological assessment tools).

Most studies attempted to understand if stressful events (or trauma) could have a role (trigger) in TTS, but findings are conflicting. As suggested by the recent Expert Consensus Document on TTS (Jelena-Rima et al., 2018), one of the key questions to answer is which role triggering factors have in the stress response of the heart. Nevertheless, the etymology of “stress” cardiomyopathy requires specific attention for the role of psychological stressor as possible etiological factor. Unfortunately, our review does not allow any conclusion by this side. The first point that warrants attention is the difference between studies drawing data from standardized psychometric tools or from retrospective assessment of medical records. Among studies based on standardized tests, none allowed any conclusion toward a role for psychological trigger events in comparison to other cardiac events (control group). On the other hand, all studies that draw data from medical records (usually based on clinical interview at admission) evidenced a role for psychological trigger events compared to controls. Obviously, this opens both to methodological and clinical considerations. From a methodological side, the use of standardized measures of assessment would lead to strongest conclusions, but in a direction making questionable the evidence of a role of psychological factors closely involved in the etiology of TTS. On the other hand, homogenous clinical observations by medical records suggest implementing further case-control studies to support the role of psychological triggers in TTS.

Some studies differentiated between “emotional” and “physical” traumas preceding TTS. Distinguishing between “emotional” or “physical” dimensions may be a critical matter, as it is very difficult to imagine any physical trauma not burdening on the emotional side. Life-threatening illnesses such as myocardial infarction or TTS may cause PTSD symptoms (Edmondson et al., 2012; Salmoirago-Blotcher et al., 2016) to testify the mutual interplay between mind and body. The unanswered question is why a person develops TTS and another one other disorders.

Summing up the various findings, we cannot prove or refute a role for psychological trigger events, differentiating what happens in patients with TTS versus those with other cardiac diseases. The role of stress in the genesis of coronary heart disease is known and relates to the sympathetic system and hypothalamic – pituitary – adrenal axis leading to increased levels of catecholamines and cortisol with a cascade of events predisposing to cardiac disease (Ndrepepa, 2017). Not finding any differences between TTS and myocardial infarction as regards to psychological triggers may delineate at some level the involvement of similar mechanisms.

Furthermore, homogeneous (albeit still limited) findings are driven from the analysis of studies evidencing a role for long-lasting psychological distress. A role for early traumatic psychological experience has been evidenced as predisposing factor for patients with cardiovascular diseases (Thurston et al., 2014, 2017; Bomhof-Roordink et al., 2015; Winning et al., 2016), and deserves more attention in future studies.

Personality was examined in a small number of studies, which consequently do not allow any clear conclusion. Personality may have a role in influencing cardiac activity, because it is related to the way people usually cope with and respond to daily stressful situations. The so-called type-A personality (described as ambitious, rigidly organized, sensitive, impatient, anxious, and concerned with time management) coined in cardiologic field (Friedman and Rosenman, 1959), continues to be a central construct in current psychosomatic practice (Fava et al., 2016) and in medical research (Lohse et al., 2017). More recently, the Type-D (distressed personality characterized by negative affectivity and social inhibition) has been associated with cardiovascular disorders (Denollet, 2000). This condition is defined by two dimensions: negative affectivity and social inhibition. Our review shows conflicting results for a role of Type-D personality, with one study (Compare et al., 2013) indicating a role for social inhibition subscale in TTS (selected according to the presence of emotional triggers) and another one (Salmoirago-Blotcher et al., 2016) evidencing a similar pattern in TTS and myocardial infarction patients. Noteworthy, we have to outline that about personality types (A, D, etc.), the existing literature is still controversial (for example, these types are not even considered in the DSM-5) and more research is further warranted in this direction. Moreover, a role for specific emotional regulation patterns in TTS patients or other cardiac events needs further research as previously speculated (Bahremand et al., 2016).

Finally, we analyzed the role of psychopathologic disorders in TTS Several selected studies tried to detect if a previous (lifetime) history of psychopathology could be predictive of TTS. Even though there were methodological disparities in the way data were collected (retrospective data by review of medical records vs. prospective assessment by standardized questionnaire at hospitalization), life-time anxiety disorders might be hypothesized to have a role in TTS. Interestingly, a recent study (Lazzeroni et al., 2018) found that only patients with pre-existing anxiety disorders were at risk of TTS triggered by emotional stressful events. This finding may help to explain why only some patients experience a stressful event as trigger of TTS.

Depression and anxiety have been associated not only with TTS (Nayeri et al., 2018), but also with elevated risk of developing other cardiac diseases (Janszky et al., 2007; Kendler et al., 2009; Gustad et al., 2014; Galli et al., 2017). A recent retrospective cohort study (Nayeri et al., 2018) found that pre-existing psychopathologic disorders (anxiety, mood disorders, and schizophrenia) were associated with an increased risk of recurrent TTS, but not to survival. It is well recognized that the comorbidity of anxiety and depression is more the rule than the exception in many chronic disorders (Fava et al., 2012; Nayeri et al., 2017) including cardiac diseases (Bahremand et al., 2016). In our opinion, the comorbid occurrence of anxiety and depression should be considered as non-specific of TTS, but a more general risk factor.

A final note on the unique study (Saffari et al., 2017) evidencing worsening of the quality of life and sexuality in TTS, aspects that merit further studies.

As the main limitation of our study, there is the impossibility to make a meta-analysis, as it was in our first intention. Unfortunately, the small number of studies and the differences in the psychometric tools, timing of observation and different design among different studies did not allow to pursuit the initial aim. As stated above, some studies adopted constructs (e.g., A or D personality) not totally supported by strong evidence (not included in DSM 5), so that any sound conclusion on this topic is not allowed and further research need to be addressed on this topic. However, the rigor of methodology we relied on (Cochrane Collaboration and the PRISMA statements) allowed to get strong results and conclusion.

In synthesis, on the basis of our systematic review we cannot evidence a clear-cut role for psychological trauma preceding TTS onset, but a possible role of long-lasting emotional distress. From the side of psychopathology, we can suggest a role for life-time anxiety disorders (more than depression), but studies are needed to clarify if differences exist with other cardiac events. For personality, we cannot conclude in the direction of specific patterns differentiating TTS from other cardiac disorders.

We need studies with stronger methodology addressing the involvement of emotional events by structured interviews conducted shortly after the onset of TTS. The timing of interviewing patients should be carefully delineated (no more than 6 months) to avoid recall bias. Furthermore, multicentre studies are warranted to recruit a large number of patients and increase sample size for this relatively rare entity. The choice of an adequate control groups needs attention, because one of the main questions is whether TTS actually differs from other cardiac disorders, as regards to personality and comorbid psychopathologic disorders.

Finally, we stress the importance of a multidisciplinary approach to TTS; such an approach should involve a collaborative process between cardiologists and clinical psychologists from the diagnosis to treatment. Evidence are accumulating on the efficacy of psychological interventions for cardiac diseases (Richards et al., 2018).

## Author Contributions

FG, FB, and SC contributed to the conception and design of the study. FG organized the database and wrote the first draft of the manuscript. FG and FB made the bibliographic research and selected papers for the systematic review (in case of doubt made confirmation with SC). FB performed the statistical analysis. FB and SC read and approved the submitted version of the manuscript. All authors contributed to the manuscript revision, read, and approved the submitted version.

## Conflict of Interest

The authors declare that the research was conducted in the absence of any commercial or financial relationships that could be construed as a potential conflict of interest.
